# Concerning Lafcadio Hearn

**Published:** 1909-05

**Authors:** 


**Concerning Lafcadio Hearn.** By **George M. Gould,** M.D. With a **Bibliography** by **Laura Stedman.** Duodecimo, pp. 416. Illustrated. Philadelphia: George W. Jacobs & Co. (Cloth, $1.50.)

In some quarters, wherein dwell the miniature-domed ones and narrow-visioned peoples, there has been breathed criticism of Dr. Gould for the penning of this volume. It has been said he was harsh in some of his statements regarding Lafcadio Hearn and his early life and personality; that he was remiss in using material concerning Hearn secured while the latter was a guest in his house. All of which is the veriest tommyrot. Those of us who knew Hearn, even casually, are familiar with his eccentricities and took him at his true value; we accepted his follies, but we did not excuse them as the vagaries of an absorbed genius, and there be records of where he was incontinently fired from a newspaper office because he publicly cohabited with a negress; and this, in spite of the fact that his work as a writer was the most brilliant and the most alluringly weird of any writing which was being done at that.

Dr. Gould did not take Hearn into his home. Whether he took him in for study purposes makes no difference to anyone and the question of the ethics of hospitality has no place in the discussion. The fact that Gould did study Hearn and has written of him is sufficient, and the result is a careful and complete analysis of one of the most complex and interesting literary characters which the world has ever produced.

Hearn was a genius; so was Byron; but no one with ladylike instincts or even gentlemanly tendencies, no matter how deeply he may revere Byron as a writer can overlook or forgive him for biting a lady in the cheek in an excess of what it has pleased some of his more deeply-steeped worshippers to consider an excess of zeal in a God-given genius. Nor can we forgive or overlook the brazen persistency of Hearn's Cincinnati love affairs.

The book under consideration gives us an honest study of Hearn from all angles. The life and letters of Lafcadio Hearn, written by Elizabeth Bisland, does the same but with this difference: that in the latter work there are rough spots which have been smoothed over with Miss Bisland's hand of gentle grace. Gould simply lifts the veil and shows the picture; and, while the Bisland book is concededly invaluable as a literary study, Gould's work is none the less so and the two together make a complete exposition of a most remarkable and fascinating character.

Gould tells as much as is known of the origin of Hearn and that much is little. It is however, fairly well established that his father was an English army surgeon and his mother a Greek. For his father, Hearn had some degree of affection; but of his mother he appears to have had little concern either as to her whereabouts or her origin. It is the first unnatural note which is struck in the life of this most brilliant and unnatural man. All through Hearn's life there ran a strain of atheistic cynicism; he revelled in the gruesome; he consorted with illiterates and depravity; he was physically somewhat brave, morally, a cringing coward; his mind, unnaturally acute was retentive to a degree, and he wandered far afield into the realms of imagery and painted word pictures of alluring elegance and sweetness then to turn gleefully to a palpitating bit of vital news and write such wondrously descriptive phrases as to make his reader stare wide-eyed at the scene that the magic of his pen conjured before the vision. He was not an educated man, as education is accepted as concerning book learning; but he was an observer and he had the power to place what he saw and repeat what he heard into such exquisite language that he was, to the reader, wholly charming. Physically, he was a bit of anatomic patchwork; his bulging eye, his weak features, his thin body were out of all proportion to his mental attainments; he walked with what has been described as a "feline tread;" soft-footed, creepy, gliding and soundless; and there was always that impression given in his attitude that he contemplated some overt act. This attitude it is quite likely may be ascribed to his intense modesty and his shrinking nature which endeavored at all times to hide his physical defects.

Hearn was not a great man in any sense of the word; he was an imitator (Gould says he was an echo) and followed closely the school of the French writers; this is apparent in all his writings; there are even strong traces of his copying of the Poe style as is evidenced by his famous report of the "Tan yard murder" in Cincinnatti; a bit of newspaper work which, while it would not be tolerated by any newspaper in the world of today was, nevertheless, a classic for vivid, if disgusting, descriptive writing. In this he described a body which had been burned in a furnace and he spake glibly, almost joyously, of burning bones and bubbling brains and bursting skull. He was an imitator of other writers' style, but he added to his imitation such imagery as made his work really marvelous and almost original.

It was in 1889 when he went to Gould's home as a guest. Here, en passant, is an illustration of the man's nature. He would sit and talk to his host for hours, and then go to his room and write Gould a long letter. He seemed better able to express himself on paper than by speech. He hated to meet strangers; he was loyal to his friends.

Just prior to the time he went to Philadelphia he was in New York and visited Colonel John A. Cockerill, then the editor of *The World* and it was during this time—the days and weeks he spent in the metropolis that the writer of this saw him during his easiest moments, for it was Colonel Cockerill who was his long time friend and mentor; it was Colonel Cockerill who really "discovered" him back in the Cincinnati days when Colonel Cockerill was city editor of the *Enquirer*. Gould tells of their first meeting. Hearn had been struggling. He wrote something for publication and went to the Enquirer office to sell the manuscript. He had not the courage to go to Cockerill's office but walked with that "feline tread" up and down the hall for hours until Cockerill came out and then, in shrinking, abashed manner offered the manuscript. Cockerill, always gentle in his dealings with strangers with manuscript took the story and read it. That was the "discovery" of Hearn as a writer and from that day dates his rise in literature. It was during the time of this employment that he wrote the famous Tan yard murder story. It was during these days, too, that he was so careless of his love wanderings that Cockerill found it necessary to discharge him from his employ in spite of the fact that he was the most brilliant newspaper writer of the day. He tried to secure a license to marry a negress. He cohabited with her openly and shamelessly.

When he visited Cockerill in New York he had done much excellent work and had been accepted by the reading public as a character of fixed reputation in literature whose beautiful language and exquisite thought were impressing themselves on the letters of the day.

Of his later works all is known; how he went to Japan, married and raised a family; of his Japanese folk-tales we are familiar and yet, through it all there runs an undercurrent of deficiency which crops out at unsuspected and unusual moments; a deficiency which gives evidence that there was in Hearn a degenerate strain which by some fortuitous circumstance stopped short of actual criminality and assumed the brilliancy of genius; for of a truth the dividing line in degeneracy which separates the criminal from the genius is exceeding fine.

That Hearn's writings had an influence on the newspaper writings of the day is apparent to one familiar with the figures and the work of the times. Of course, natural advancement in methods had wrought a change in the basic elements of descriptive work; but the famous Tan yard murder story, written years agone by Hearn, that and the wonderful deductive powers of Dupin, were the inspiration for a somewhat famous news story written in New York in the late eighties shortly after the visit of Hearn to Cockerill, the tale of which is interesting. A dismembered male body was discovered in a trunk in Baltimore, and the head was missing. Among the newspapermen who went to Baltimore and came back with the fragments of the murdered one, was a newspaper man who had been a student of Hearn's methods and of Poe's. He wrote a story descriptive of the manner in which the man had been murdered and the body dismembered. A few days later when Unger was arrested for the murder of August Bolle and confessed, the confession and the newspaper description, written before the body had been identified, were with few inconsequential exceptions, almost identical.

Gould's book on Hearn is a remarkable study of a remarkable man, and it is an invaluable addition to literature. Hearn was great in a literary sense only; and because he was and because his influence on literature is lasting we may be content to step through the mire of his selective individuality that we may reach the flowers of his fancy. N. W. W.

